# Sharing of cultural values and heritage through storytelling in the digital age

**DOI:** 10.3389/fpsyg.2023.1104121

**Published:** 2023-02-21

**Authors:** Çağın Zort, Esra Karabacak, Şevket Öznur, Gökmen Dağlı

**Affiliations:** ^1^Faculty of Art and Sciences, Near East University, Nicosia, Türkiye; ^2^Faculty of Education, Kyrenia University, Kyrenia, Türkiye

**Keywords:** cultural values and heritage, digital age, education, online community, storytelling

## Abstract

**Introduction:**

Sharing cultural values in this digital age for young generations who are digital natives is highly important and, in this respect, the aims of this study are to evaluate experts’ opinions on sharing cultural values in this digital age based on their experience, the roles of educators and families with respect to the sharing of cultural values through storytelling in the digital age, and also capture how cultural values can be explained with metaphors.

**Methods:**

A focus group interview was conducted with teachers and vice headmasters from public primary and secondary schools in the Northern part of Cyprus within the age range of 30-50 years that are considered to be experts based on their 10 years and above of teaching experience. Data were analyzed through line-by-line coding to create themes.

**Results:**

Findings revealed that cultural values are eroding, and in sharing cultural values with storytelling in the digital age, the roles of educators and families are essential. Cultural values are the treasures and mirrors of society that should be preserved and transmitted to the younger generations and this can be accomplished through participation in digital platforms, and when such participatory cultural heritage projects are planned with a community-oriented background and human-centered computing concentration.

**Discussion:**

This research sheds a light to indicate the importance of the storytelling approach for sharing cultural values and heritage. It is significant to address the merits of technology in transferring cultural values and heritage. In addition to this, this study is limited to one specific context that can be further explored as a cross-cultural analysis.

## Introduction

The future of institutions and countries is related to protecting, protecting, and sharing their cultural values and heritage. The most basic elements that sustain an institution are the values it creates, and the norms, stories, and cultural ties they share together to create values. In the formation of cultural ties, it is necessary to be aware that the communication, interaction, and sharing process should be effective and be accepted by everyone (Adam-Troian et al., 2021) because the manner in which we talk and associate with one another formulate culture, whether in social orders, organizations, or relationships (Hendrith, 2018) and the sharing process that can be aided by culture sharing activities that are specifically significant for young learners (Hastings, 2018) most especially in this digital age. The digital age is now and then denoted as the information age (Chiulli, 2020) where nearly all if not all activities are dependent on technology.

In the digital age where digital effectiveness is required (Fonseca and Domingues, 2017), culture has been described by Goran et al. (2017) as one of the most important self-reported challenges to digital effectiveness. Gallivan and Srite (2005) noted that one steady assumption behind most cultural explorations within the domain of information systems shows that culture does not change and remains stagnant. Despite the fact that in the short term, cultures are to a great extent stable (Pliskin et al., 1993), cultural modifications happen because of a different set of issues like the international media, immigration, and technological and social patterns (Ford et al., 2003; Gallivan and Srite, 2005; Slimbach, 2005). Salehan et al. (2018) added that the sociocultural perspective of globalization acknowledged the incessant modification of cultural values and heritage across the globe. According to Heilbroner (1994), technological determinism theory suggests the technology of any society will determine the advancement of its cultural values, heritage, and social construct. In education, the place of the teacher is more fluid and less obvious in this digital age because educators’ roles are no more restricted to their customary responsibilities (Grand-Clement et al., 2017).

Several extant studies like Mabovula (2011), Nicu et al. (2020), Wahab et al. (2012) have confirmed the rapid erosion of cultural values and heritage, and one of the major antidotes is to continue to encourage the younger generations to learn about their cultural values and heritage because culture is learnable (Spencer-Oatey and Franklin, 2012). Cultural intelligence which has been described by Peterson (2010) as the skill used to work and relate adequately in culturally diverse circumstances, has also been acknowledged as a must-have 21st-century skill to lead and work (Cultural Intelligence Center, n.d.). To highlight the topical issue of eroding cultural values and heritage, Harvey (2019) went as far as raising global consciousness on the erosion of cultural values and heritage from the perspective of climate change by stating that the larger part of the debate on contemporary issues like climate change puts more attention on physical consequences, for example, increased cruelty and recurrence of tempests, the rising level of the sea, higher insurance rates, diminishing values of property, and decreasing farmable environment. Be that as it may, the intangible like cultural consequences of climate change is not been regularly discussed and these incorporate the loss of ways of life and customary practices. Harvey (2019) went further that the loss of ways of life and customary practices can prompt decreased social cohesion and general mental health problems in society on the grounds that a person’s way of life (i.e., culture) is firmly connected with that person’s sense of belonging and identity in the community. Moreover, another obvious fact is that cultural heritage is intimately connected and dependent on people’s relationship with their indigenous habitat. This implies that empirical explorations should be geared toward the transmission of cultural values and heritage from one generation to another, most especially in this digital age *via* storytelling.

If culture is learnable (Spencer-Oatey and Franklin, 2012), then it is teachable and one of the strategies to teach and transfer culture is storytelling. Storytelling, as part of the most significant and dynamic parts of human nature and culture (Frank, 2022), coupled with the fact that contemporary learners are majorly digital natives, is essential to integrate the application of technology in this digital age to preserve cultural values and heritage through storytelling. According to Benjamin (2006), poorer language skills are the consequence of cultures with little storytelling, and a decent storyteller with creative speech trends has the tendency to impact an entire group of imitators. Barger (2001) added that it is impossible to forget vocabulary words that are kept alive in stories. As affirmed by Wittgenstein (2013) that language has a significant impact on culture, Benjamin (2006) concluded that storytellers among humans create and preserve the culture of every society.

Society should share and multiply its existing values in order to make its political and economic power sustainable in its geography, and it should protect its cultural values and heritage so that the cultural structure can be solid. Of course, trained manpower, which we call intellectual capital, can add cultural value and richness in every field is trained in a society that will keep the cultural structure and values of society alive and sustaining required (Mariati et al., 2021).

In order to create social success and ensure the visibility of the country, opportunities for intellectual capital, employment, motivation to work and life, self-realization, and development are very important. The dignity and respect attributed to every individual who carries a society to the future is the true mirror of society. Therefore, it is necessary for every individual living in the society to approach the literature of the country as a part of well-being and to recognize its historical, geological, and geographical values in order to ensure the integrity of the soul and body and to move the future forward with strong cultural values (Turdiyev, 2021). Well-being is defined by being aware of existence and its past and accepting that it is a holistic value. The literature of societies and the values of intellectual people who grow up in societies contribute to the well-being of each individual. Thus, the sharing of cultural values and heritage, their transfer from generation to generation, and most importantly, their inclusion in education programs through the education system predicts that cultural heritage will gain a sustainable structure (Yoo et al., 2021).

Considering the fact that information technologies have gained momentum and will be effective in every sector after the pandemic process, the existence of technology platforms where sharing and interaction are planned correctly has gained great importance. Online meetings and digital books both provide fast sharing and make these shares visible in the world. Therefore, technology-supported sharing of cultural values and heritage is necessary not only for education but also for the recognition and survival of a country in many areas such as tourism and the economy (Hisa et al., 2022; Martins et al., 2022).

### Research aims and significance

The aims of this study are to evaluate experts’ opinions on the transmission of cultural values based on their experience in this digital age, the roles of educators and families with respect to the sharing of cultural values through storytelling in this digital age, and also capture how cultural values be can be explained with metaphors. This study will assist in dissecting educators’ opinions on storytelling in the digital age that will enhance sharing of cultural values to transfer and preserve cultural values and promote cultural intelligence that is required to strive in a 21st-century multicultural setting. It will add to the existing literature on culture and storytelling from experts’ perspectives. It will aid in understanding the significant roles of families with respect to the sharing of cultural values.

To achieve the aim of this study, the following research questions were posed;

1. What are the experts’ opinions on sharing cultural values based on their experience in this digital age?
2. How can cultural values be shared in this digital age through storytelling?
3. What are the duties of educators regarding the sharing of cultural values?
4. What are the roles of families in this digital age regarding the sharing of cultural values?
5. How can cultural values be explained with metaphors?

## Literature review

### Cultural values and heritage

Culture as a concept has been a hard nut term to describe, to the extent that American anthropologists Kroeber and Kluckhohn as far back as 1952 basically studied definitions and concepts of culture and came up with 164 unique definitions (Spencer-Oatey and Franklin, 2012). To address this disparity, Apte (1994) summed up the issue as follows: In spite of 100 years of attempts to define culture effectively, there has been no consensus among anthropologists with respect to the nature of culture in the early part of 1990. For concision purposes, this study will stick with the definition of Spencer-Oatey (2008:3);

*“Culture is a fuzzy set of basic assumptions and values, orientations to life, beliefs, policies, procedures, and behavioural conventions that are shared by a group of people, and that influence (but do not determine) each member's behaviour and his/her interpretations of the 'meaning' of other people's behaviour.”*

According to Spencer-Oatey and Franklin (2012), as humans relate with one another, culture is learned from individuals we interact with, and seeing how grown-ups respond and converse with infants is a superb method for seeing the genuine emblematic transmission of culture among individuals. Ishii and Eisen (2021) added that cultural values and heritage are entrenched in day-to-day practices, routines, interaction styles, relational talks, and public symbols in which individuals connect unwittingly and regularly. The transmission of cultural values, heritage and standards from one generation to the other through process guarantees the continuity of customs within a gathering of individuals (Jegatheesan, 2015). Acquisition of a culture is something that one learns (Spencer-Oatey and Franklin, 2012) and this shapes the beliefs, traditions, and attitudinal agreements of the whole society (Jegatheesan, 2015). The study of Zhu and González Martínez (2022) gives insights into cultural heritage as an effective tool for redevelopment and value creation. In addition, the study of Lawton et al. (2022) becomes an example of how digital tools increase the values of culture.

### Storytelling in digital age

Bowles (1995) and Koch (1998) acknowledge the significance of storytelling as it has been utilized for a really long time as a strong driver for entertainment, education, communication, and transmission of cultural values and heritage. Park (2001) noted that storytelling is a fundamental and special element of the human experience. This scholar went further that irrespective of medium (poem, drama, novel, myth, or fairy tale), stories are delighted in by everybody. Although stories, whether through poetry or fiction, were archeologically portrayed; the contemporary methods of communication incorporate printing, radio, television, film, and digital platforms (Davidhizar and Lonser, 2003). Davidhizar and Lonser (2003) added that stories have offered a means for keeping cultural values and heritage. Adults utilized storytelling as a method for interpreting history for younger generations and the history of every family is frequently transmitted through diaries, books, or stories about the family. It should be valued that consistent storytellers will generally share stories that uphold their stand and positive self-view. Critical issues bordering on slavery, Hiroshima, or Holocaust might be shared through storytelling according to the interesting point of view of the individual sharing the story (Bailey, 2011). Nevertheless, stories are in some cases impartial (Davidhizar and Lonser, 2003; Robin, 2008). Recently, the utilization of novel technologies in school systems has expanded globally as a result of novel inventions like digital cameras, laptops, mobile phones, software, and the development of digital platforms through the internet which have opened up to teachers to become attached to the digital world (Smeda et al., 2014). Quah and Ng (2022) give an insight into a review of storytelling which reflects that digital storytelling tools are useful to promote the required 21^st^-century skills, particularly literacy skills, ICT literacy, collaboration, and creativity. It is suggested that digital storytelling tools need to set the stage for reflective practices for higher transformative learning goals.

Several studies have discussed the significance of technology from diverse fields of study such as education (Altınay et al., 2016; Altınay-Gazi and Altınay-Aksal, 2017; Olszewski and Crompton, 2020; Hawkridge, 2022), health (Singh et al., 2020; Sun et al., 2020; Bokolo, 2021), engineering (Longo et al., 2020; You and Feng, 2020), etc., and positive impacts of technology advancement that has caused a persistent increase in human dependency on technology (Bicen and Adedoyin, 2022), thereby causing significant modifications to how humans relate and live (i.e., culture; Salehan et al., 2018). The effect of new technologies in instructional settings has been for the most part positive due to opportunities provided by new technologies for teachers to upgrade their insight and abilities, and in this way improve the quality of education (Isisag, 2012; Costley, 2014; Kuimova et al., 2018). Scholars (Paino, 2009; Parsons and Taylor, 2011; Gunuc, 2014) have observed that learners’ motivation, engagement, and achievement are improved through the utilization of these new technologies.

Luckily, contemporary young learners have accessible opportunities that digital immigrants never envisioned when they were young because of novel and stimulating learning experiences that have graced the forefront of education generally on account of the development of instructional technology and the more extensive accessibility of instruments and strategies through digital learning platforms (Hastings, 2018). From inception, one of the most significant and dynamic parts of human nature and culture is storytelling, and it has been changing over time due to technological advancement but the purpose of storytelling which is to pass messages has remained constant (Frank, 2022).

Storytelling in this digital age on digital platforms has been described by several scholars, with a term like digital storytelling but one consistent fact that cannot be negated is the link between storytelling, digital technologies as tools, and the digital platforms where stories are shared (Miller, 2014). Stories are told *via* several digital platforms like social media, and devices such as interactive books, video games, etc., and they provide opportunities for users to interact with stories. Recent studies put an emphasis on digital platforms and story-telling for cultural values and heritage in the Covid-19 period. For example, the study of Ginzarly and Srour (2022) study on cultural heritage in an online context. The storytelling approach has increasing importance in educational practices as part of the value of education. With digital transformation, digital storytelling can be possible in different forms on a personal and emotional level. Storytelling is developed for also cultural heritage in various contents. Storytelling for cultural heritage focuses on specific topics from details to create a whole picture. Cultural and value-based stories need to be facts that could be rich in content, informative, and clear on digital platforms.

## Methodology

### Research design

This study uses the qualitative research design. Qualitative research reflects an in-depth emic perspective-based research process based on the power of meanings, based on detailed facts, experiences, and opinions. In this study, a research process was carried out with detailed enlightenment and interpretation in line with expert opinions and experiences (Creswell, 2014).

### Study participants

Participants of this study are teachers and vice headmasters from public primary and secondary schools in the Northern part of Cyprus within the age range of 30–50 years that are considered to be experts based on their 10 years and above of teaching experience. Purposive sampling was applied as the sampling method because the study is only interested in experts’ opinions, and a threshold of more than 10 years of teaching experiences in primary school and secondary school was adopted, in which 8 females and 9 males totalling 17 experts who met the established threshold voluntarily participated in the study after informing them of their right to withdraw their participation at any time and for any reasons.

### Research instrument and data collection process

In this study, a focus group interview was used as the research instrument for data collection with the aid of unstructured interviews. Participants were contacted for a seminar on the transmission of cultural values and heritages in the digital age, and they were informed about the purposes of the study, their consent to participate, and as well as their right to withdraw their consent for any reason at any time. Unstructured interview questions were posed to the participants after the seminar, the interview session was recorded and transcribed.

### Data analysis

The transcribed data was subject to line-by-line coding as prescribed by Chenail (2012) for qualitative data thematic content analysis to establish the frequency of ideas and themes based on Anderson’s (2007) thematic content analysis for the descriptive presentation of qualitative data.

## Findings & Discussions

### Experts’ opinions on sharing cultural values and heritage based on their experience

To answer the first research question on the experts’ opinions on sharing cultural values and heritage based on the participants’ experience, their responses were coded to establish the themes in Table 1.

**Table 1:** Sharing of cultural values and heritage based on experts’ experience.

Themes	Frequencies (N)	Percentage (%)
Cultural values and heritage began to be lost all over the world, including in our country.	8	47
Cultural values and heritage should be transferred to new generations.	11	64
Cultural values and heritage need to be protected	12	70
Keeping cultural values and heritage alive for generations	9	52
More emphasis on cultural values and heritage in schools	14	82

The following are some of the excerpts from the experts’ opinions based on their experience;

*P1*: “Unfortunately, I think we are starting to lose our cultural values in the global world. When we use our own culture outside of certain areas, we are seen as a reactionary structure by the new generation”.

*P2*: “Despite the changing social structure today, culture shows its effect from the birth of people. For this reason, on social media platforms, individuals can share their cultural values through every opportunity”.

*P3*: “Through storytelling, I try to transfer our cultural values to new generations as much as I can”.

*P4*: “We have a culture that has become a part of life. We live with many pieces of culture in daily life. It is a way of life, from food to folklore, to oral entertainment like storytelling”.

*P5*: “A cultured person, regardless of education level, the transfer of culture to the individual is evident from the family. Even sitting or standing, it symbolizes both the education and the culture he has received. Even if one of our family elders is sitting or trying to stay while sitting, I say, grandfather, father, or mother. In my opinion, I think that another important concept in cultural values is respect”.

According to the opinions of the participants, it can be said that cultural values and heritage have begun to erode, most especially in North Cyprus and this also confirms the findings of extant studies like Mabovula (2011), Nicu et al. (2020), Wahab et al. (2012) from other countries. Furthermore, participants suggested that cultural values and heritage should be preserved, kept alive, and transferred to new generations as earlier recommended by Kruft and Gamber (2021) and Bitzer et al. (2021), and more importance should be given to the issue of cultural values and heritage in schools. This implies that since culture is learnable (Spencer-Oatey and Franklin, 2012), educators should concentrate on teaching cultural values and heritage in schools, to protect, preserve, and transmit to the younger generations. This can be accomplished through participation in digital platforms, and when such participatory cultural heritage projects are planned with a community-oriented background and human-centered computing concentration (Liew et al., 2022).

### Sharing of cultural values and heritage through storytelling in the digital age

To answer the second research question on how cultural values can be shared through storytelling in the digital age, participants’ responses were coded to establish the themes in Table 2.

**Table 2:** Sharing of cultural values and heritage through storytelling in the digital age.

Themes	Frequencies (N)	Percentages (%)
Introducing our own cultural values and heritage to guests from foreign countries	6	35
Sharing the cultural values and heritage we learned from our elders with our friends	13	76
Focusing on sister school projects from foreign countries	10	58
Allocating time for more cultural values and heritage, organizing activities on weekends	7	41
Situations related to cultural values and heritage for our citizens living in foreign countries	4	23

The following are some of the excerpts from the participants’ responses on how cultural values and heritage can be shared through storytelling on digital platforms;

*P1*: “We hosted a German citizen coming from Germany online and I introduced him to our village life and our own customs prior to his arrival by telling him stories about our community”.

*P2*: “One day I gathered with my friends, I said a word I learned from my grandmother at our meeting, and when there is a suitable environment, my friends say it and I see that I pass it on to them”.

*P3*: “I took part in this team when we hosted teachers and students as a school for the sister school project from Istanbul. We shared live broadcast of the event via social media platforms and many people joined the live broadcast to learn more about our cultural values. We offered the food and drinks of our culture to them and gave them as gifts. We also showed them our cultural clothes and music and made them listen to it and some significant stories attached to those cultural clothes, music, foods, and drinks”.

*P4*: “The person who is our guest has some surprises about drink, for example, as a drink, and when I tell him his solutions to colds, he is stunned”.

*P5*: “It has become a tradition in our country to have a barbecue with family elders on the weekends. Usually, we try to have a family barbecue on weekends, video record the whole process and share online with family and friends and we intend to continue this tradition”.

Based on the opinions of the participants on sharing cultural values through storytelling in this digital age, it can be underlined that telling stories to introduce our own cultural values and heritage to the guests coming to our country from foreign countries *via* digital platforms has an important value in the spread and permanence of cultural values and heritage. Using stories to share what people learn from older generations and friends is of great importance in transferring our values to the younger generations and not losing the values over time. This is in consonance with Barger’s (2001) and Benjamin’s (2006) findings that storytellers among humans create and preserve the culture of every society. Sharing of cultural values and heritage through events where people endeavor to tell stories associated with their clothes, foods, music, etc. will play an important role in the globalization of cultural values and heritage based on the instance of stories given to focus on sister school projects from foreign countries (Barnett and Woods, 2021; Toker Gökçe, 2021). It can be evaluated according to the opinions of the participants that especially among families, spending more time on cultural values and heritage and doing activities on weekends, sharing pictures and video recordings of those events online will play an important role in keeping cultural values and heritage alive in this digital age. One important factor that can emanate and is worthy of note is the online discourse community that pictures and videos recorded for sharing values on digital platforms create after uploading them based on the position of Seraj (2012) where the scholar opined that digital platforms have turned into an indispensable hotspot for knowledge creation and utilization while online communities have ended up being the new type of socialization stages for satisfying specific requirements like acquiring and providing information, sharing cultural values and heritage, experiences, etc.

### Duties of educators on sharing of cultural values and heritage in the digital age

To answer the third research question on the duties of educators in sharing cultural values and heritage in the digital age, participants’ responses were coded to establish the themes in Table 3.

**Table 3:** Duties of educators regarding sharing cultural values and heritage in the digital age.

Themes	Frequencies (N)	Percentage (%)
Customary traditions need to be included and explained more in some courses.	3	17
Educators should carry the culture they live in and connect the subjects with culture at every moment of education and transfer them to students from a young age.	8	47
Teachers should share a multi-cultural structure formed in the classroom with each other and take time to share their own experiences.	3	17
Giving more importance to folk dances in schools, giving due importance to the music of our culture.	5	29
Increasing catchy and instructive activities by giving assignments to students in the form of theater with pictures or compositions.	4	23

The following are some of the excerpts from the participant’s responses on the duties of educators in sharing cultural values in this digital age;

*P1*: “I think we should include and explain our customary traditions more in some lessons”.

*P2*: “Educators should carry the culture they live in and connect the subjects with culture at every moment of education and transfer them to students from a young age. Teachers should take care to share a multi-cultural structure consisting of the classroom with each other and to convey their own experiences”.

*P3*: “More importance should be given to folk dances in schools, and the music of our culture should be given due importance”.

*P4*: “Folk dances should be given more importance in schools, and the music of our culture should be given due importance”.

*P5*: “Educators, after instilling the culture and the culture inherited from the family, our teachers should work towards further development. In order to make this memorable and instructive by giving assignments to the students in the form of theatre with sample pictures or compositions, the educator and the learner can help each other to settle the concept of culture together”.

Based on the opinions of the participants on the duties of educators in sharing cultural values in this digital age, customs and traditions should be included and explained more in the classrooms. Educators should carry the culture they live in and it is understood that they should take serious care in connecting the subjects with the culture at every moment of education and transferring them to the students from a young age. It is also evident that multicultural structures formed in the classroom should be shared with each other and educators should take time to share their own experiences. By doing all these, they might have the potential to improve the cultural intelligence of students because cultural intelligence has been highlighted as of must-have 21st-century skill to lead and work effectively in a culturally diverse world (Cultural Intelligence Center, n.d.). It is clear that attention should be given to the importance and application of folk dances in schools with videos of folk dancers that educators cannot bring to class due to several factors like distance, finances, etc., but their videos are on digital platforms to teach students based on the significant relationship between music and culture (Campbell, 2021). Additionally, a close relationship has to be established between the students and educators to enhance the transmission and preservation of cultural values and heritage in schools where assignments will be given to the students to write compositions about the culture, and act in dramas that showcase their cultural values and heritage. These compositions and dramas can be shared on digital platforms for others to access. This also confirmed the position of Sleiman (2021) on using engaging activities like drama, writing compositions, pictures, etc., for sharing cultural values. Participants’ responses correlate with the recommendation that educators should consider collaborative design, sharing, and interpretation of digital cultural heritage content (Koutromanos et al., 2022), to eradicate or minimize divergent views on cultural value content (Kucirkova and Flewitt, 2020).

### Duties of family on sharing of cultural values and heritage in the digital age

To answer the fourth research question on the duties of family in sharing cultural values in the digital age, participants’ responses were coded to establish the themes in Table 4.

**Table 4:** Duties of family regarding sharing cultural values in the digital age.

Themes	Frequencies (N)	Percentage (%)
While raising their children, families should raise them with the habits of food and clothing specific to their own nation.	4	23
The family should teach their children by keeping their culture alive with digital resources used in raising them.	6	35
First of all, the family should respect and love the elders, hug and love the little ones, treat everyone well, protect the animals, and not harm all living things.	3	17
Sharing the importance of our family ties, our island, and the cultural values that make us who we are on digital platforms.	2	11
Protecting cultural values and heritage in the family and acting in this way	5	29

The following are some of the excerpts from the participant’s responses on the duties of family in sharing cultural values and heritage in this digital age;

*P1*: “I think that when raising their children, most especially with digital materials like instructional videos and images, families should raise their own with nation-specific food, clothing habits, etc. Otherwise, societies that have lost their own culture are doomed to disappear from the stage of history”.

*P2*: “The family should teach their children by keeping the culture they have alive and ensure that it is embedded in every digital material used in raising them”.

*P3*: “Family should teach children with digital materials that propagate respect to elders, love, hugs and love our little ones, treat everyone well, protect animals, and not harm all living things”.

*P4*: “Families should stay away from digital resources that polarize and reflect culture based on political views. I think families should take it as their duty to share humanity, hard work, the importance of family ties, and the cultural values that make our island and us who we are when using digital platforms for teaching cultural values”.

*P5*: “Families need to protect our culture. Children's first role models are their parents. Protecting the cultural values in the family and acting in this way will bring up a new individual who has cultural values through online dramas that tell stories about self-respect and respect for the people around him.”

Participants’ responses placed significant responsibility on families to raise their children with food and clothing habits that are specific to their own nation, within the scope of the digital materials used in raising children. These digital materials are both extensively transient and everlasting compared to prior means of communication most especially when raising children (Lewis, 2014). The issue of compassion, care, and sensitivity in matters such as respect for the elders, love, hugging and loving the little ones, treating everyone well, protecting animals, and not harming all living things was raised by participants when using the digital platforms and resources to share cultural values and this resonates the idea of Sukhera and Poleksic (2021) that since technology is both necessary and unavoidable, it should be embedded in compassionate care. This implies that compassion, respect for the elders, love, hugging and loving the little ones, treating everyone well, protecting animals, and not harming all living things should be top priorities for families in selecting digital materials or environments for sharing cultural values with their children. It is understandable based on participants’ opinions which conforms with Albert et al. (2021) conclusion that families have great responsibilities in sharing the importance of family ties and the cultural values that make society and the people who they are. According to the results of the research, it can be said that families should raise their children with food and clothing habits specific to their own nation, within the scope of family strikes regarding the sharing of cultural values and heritage. It is understood that families should take the necessary care to teach their children by keeping the culture they have alive.

### Explaining cultural values and heritage with metaphors

To answer the fifth research question on how cultural values and heritage be explained with metaphors, participants’ responses were coded to establish the themes in Table 5.

**Table 5:** Explaining cultural values and heritage with a metaphor.

Themes	Frequencies (N)	Percentage (%)
Mirror of society	5	29
Cultural values and heritage are like home	3	17
Library	2	11
Cultural values and heritage are like a treasure	5	29
Similar to region countries map	2	11

The following are some of the excerpts from the participant’s responses on how to explain cultural values with a metaphor;

Explaining Cultural Values and Heritage with a Metaphor,” and some of the participant’s views are presented below in light of this dimension.

*P1*: “It is the mirror of the society”.

*P2*: “We can compare society to a house. If we see individuals as bricks, we can say that culture is the cement that unites them”.

*P3*: “I am making my example by analogy with the Map of region countries because just as each city is given a license plate, each person comes to life in a different family and begins to breathe the breath of that family”.

*P4*: “Library: The place and section of all books are different. Thus, an order is formed in the library and even someone who has not been to the library before can easily find the book they want. There is no confusion in the culture because he knows where and how to behave in the same way”.

*P5*: “We can like cultural values to a treasure. I think that the most important treasure of a society is its cultural values”.

In the context of explaining cultural values with a metaphor, it is understood that the reflection of the values of the society coincides with the cultural values and this shows the relationship through the expression that they are the mirror of society. According to Liu (2001), names are a significant part of culture because when people’s names are explained from philosophical, historical, and etymological perspectives, the natural and social environment of these people psychologically reflects. It can be said that each brick of the house corresponds to each value of the society within the context of cultural values and the metaphor of society being like home. Cultural values are likened to a map and they stated that the lives in each city and settlement unite and form the cultural values of the society. In addition to these, cultural values are likened to a library, the location and sections of all books are different, and thus an order is formed in the library, and even a person who has not been to the library before can easily find the book he/she wants. In this context, since the members of the society know where and how to behave in the same way, this can automatically prevent the formation of confusion (Onecha et al., 2021). Participants were of the opinion that the most important treasure of a society is its cultural values, especially by comparing its cultural values to a treasure. Cozzani et al. (2017) evaluated the extent technology can have an impact on the education and preservation of intangible cultural heritage through a European Community-funded project called i-Treasures, and the results indicated that the project is having valuable impacts on the preservation and transmission of cultural values and heritage. This implies that participants’ position on equating cultural values and heritage to treasures is not out of place and these cultural values can be preserved and transmitted to the younger generation through technology.

## Conclusion

The aims of this study are to evaluate experts’ opinions on sharing cultural values and heritage on the digital platform based on their experience, the roles of educators and families with respect to the sharing of cultural values through storytelling in digital platforms, and also capture how cultural values be can be explained with metaphors in digital platforms. From the opinions of the participants in comparison with the findings of extant studies from other countries, it is evident that cultural values have begun to erode not only in North Cyprus but globally. Hence, the need to protect, preserve, keep alive and transfer cultural values to new generations can be achieved through digital storytelling. This can be accomplished through participation in digital platforms, and when such participatory cultural values and heritage projects are planned with a community-oriented background and human-centered computing concentration. It can be underlined that storytelling can be applied to introduce cultural values and heritage to foreigners, friends, and younger generations to spread, transmit, and preserve cultural values.

Cultural values and heritage are part of the sustainable development goals which the education system needs to cover for the lifelong learning philosophy. In terms of generic skills development of the new generation, knowing more cultural values and heritage with digital transformation need to be internalized within the system. It is the global perspective to consider dignity and solidarity within the culture to practice sharing values and heritage from generation to generation. Therefore, the educational management perspective needs to consist of cultural values and heritage, compatible with sustainable development goals for the future of education and the welfare of society.

Educators and families also have important roles in transmitting and preserving cultural values by integrating cultural values and heritage into their activities in the classroom and digital platforms. In the context of explaining cultural values with a metaphor, it is understood that the reflection of the values of the society coincides with the cultural values and shows parallelism, by expressing that they are the mirror of society. It can be said that each brick of the house corresponds to each value of the society within the context of cultural values and the metaphor of society being like home. Cultural values and heritage are likened to a map and they stated that the lives in each city and settlement unite and form the cultural values of the society.

### Limitations and recommendation

Despite the fact that the findings of this study are essential and also have the possibility of influencing practice and policy, generalizations cannot be guaranteed based on the fact that the sample size is small and participants are from only public primary and secondary schools in North Cyprus. Further studies can focus on the inclusion of faculty members from universities to have all-encompassing expert opinions. Participants from other countries can also be included for comparative analysis.

## Data availability statement

The original contributions presented in the study are included in the article/supplementary material, further inquiries can be directed to the corresponding author.

## Ethics statement

The studies involving human participants were reviewed and approved by Near East University Ethics Committee Board. The patients/participants provided their written informed consent to participate in this study. Written informed consent was obtained from the individual (s) for the publication of any potentially identifiable images or data included in this article.

## Author contributions

ÇZ and GD: conceptualization. ÇZ: methodology, formal analysis, data curation, writing—original draft preparation, writing—review and editing, and funding acquisition. ŞÖ: software, validation, and supervision. EK: investigation. ÇZ and EK: resources. ÇZ, ŞÖ, and GD: visualization. GD: project administration. All authors contributed to the article and approved the submitted version.

## Conflict of interest

The authors declare that the research was conducted in the absence of any commercial or financial relationships that could be construed as a potential conflict of interest.

## Publisher’s note

All claims expressed in this article are solely those of the authors and do not necessarily represent those of their affiliated organizations, or those of the publisher, the editors and the reviewers. Any product that may be evaluated in this article, or claim that may be made by its manufacturer, is not guaranteed or endorsed by the publisher.
